# Smoking Behaviour and Beliefs About Smoking Cessation After Bariatric Surgery

**DOI:** 10.1007/s11695-020-04907-4

**Published:** 2020-08-16

**Authors:** Paula J. D. Wolvers, Oranos Ayubi, Sjoerd C. Bruin, Barbara A. Hutten, Dees P. M. Brandjes, Eelco W. Meesters, Victor E. A. Gerdes

**Affiliations:** 1 Department of Vascular Medicine, Amsterdam UMC, University of Amsterdam, Meibergdreef 9, 1105 AZ Amsterdam, Netherlands; 2 Department of Surgery, Spaarne Gasthuis, Spaarnepoort 1, 2134 TM Hoofddorp, Netherlands; 3 Department of Clinical Epidemiology, Biostatistics and Bioinformatics, Amsterdam UMC, University of Amsterdam, Meibergdreef 9, 1105 AZ Amsterdam, Netherlands; 4 Department of Internal Medicine, Spaarne Gasthuis, Spaarnepoort 1, 2134 TM Hoofddorp, Netherlands

**Keywords:** Bariatric surgery, Smoking, Smoking cessation, Beliefs, Weight loss, Long-term outcomes, Comorbidities

## Abstract

**Purpose:**

Currently, bariatric surgery is the most effective intervention for treating morbid obesity and its complications. Smoking cessation is likely to improve smoking-related comorbidities and decrease postoperative complications. This study evaluated the smoking behaviour and thoughts about smoking cessation of patients more than 18 months after bariatric surgery.

**Materials and Methods:**

A cross-sectional study was performed in patients who underwent bariatric surgery from July 2012 to December 2013. A questionnaire was used to evaluate smoking status, thoughts about the health benefits of cessation and characteristics of previous quit attempts in current and former smokers. Finally, actual bariatric surgery outcomes were evaluated in current, former and never smokers.

**Results:**

Six hundred nine patients (response rate 52.0%) were included. Of them, 101 (16.6%) patients were current smokers, 239 (39.2%) former smokers and 269 (44.2%) patients were lifetime never smokers. Compared with former smokers, current smokers were less aware of the beneficial effects of smoking cessation on their general health; 66.4% of the former smokers thought smoking cessation would be much better for general health, compared with 20.6% of current smokers. Total weight loss was 2.8% higher in current smokers compared with former smokers. Actual long-term bariatric surgery outcomes were not significantly different between the groups.

**Conclusion:**

Despite advice to quit smoking and temporary quitting before surgery, a considerable group of bariatric surgery patients continues smoking after surgery. These patients were less aware of the beneficial effects of smoking cessation. This study emphasizes the need for better strategies to increase the number of successful cessations.

## Introduction

Currently, bariatric surgery is the most effective intervention for treating morbid obesity and obesity-related complications. It contributes to long-term weight loss, improvements in comorbidities and reduction in mortality [1–3]. Smoking is suggested to be a modifiable preoperative risk factor that significantly increases the incidence of postoperative morbidity and mortality in bariatric surgery [4–7]. Several studies have shown that smoking cessation is associated with improved smoking-related comorbidities and decreases the incidence of postoperative complications [4, 5, 8–11]. Therefore, patients are urged to quit smoking before undergoing bariatric surgery and continue this after the operation in our centre.

However, some aspects could negatively affect a successful smoking cessation and its duration before and after surgery, which could impact short- and long-term outcomes of bariatric surgery. Examples are the concern of weight gain after smoking cessation, inadequate beliefs about the health consequences of smoking, doubts about the benefits of cessation, previous unsuccessful attempts and the experienced lack of support [12–19].

A few studies have evaluated the smoking cessation rates after bariatric surgery, which ranged from 0 to 20.7%. They have demonstrated that smokers who quit (long) before surgery started again after surgery [20–22]. These disappointing percentages could partly explain why several studies showed no difference in smoking prevalence before and after bariatric surgery or even higher prevalences of smoking after surgery [23–25].

In this study, we evaluated the smoking status of patients at least 18 months after bariatric surgery using a questionnaire. Secondly, we compared smoking history and thoughts about the effects on health outcomes between patients who stopped smoking and those who continued. Finally, we evaluated actual bariatric surgery outcomes in the subgroups defined by smoking status.

## Methods

### Design, Setting and Population

A cross-sectional study was performed in a high volume bariatric surgery centre where bariatric surgery has been performed since 2007, from July 2015 until September 2016. The hospital has been accredited as Bariatric and Metabolic Surgery Centre of Excellence by the European Accreditation Council for Bariatric Surgery in 2013 [26]. At least 18 months after surgery, patients who underwent primary or revisional bariatric surgery between the 1st of July 2012 and 31st of December 2013 were eligible for the current study. Preferably, patients were approached when attending the clinic for routine follow-up visit. If patients did not show up, then they were contacted by mail, e-mail or telephone if details were available. All patients in this cohort were advised to stop smoking at least 2 weeks prior to the surgery. They received regular follow-up visits after surgery, after the visits of the first year at least one visit every year.

### Ethical Approval

This study was performed in accordance with the ethical standards of the Helsinki Declaration. All patients provided written consent. The Institutional Review Board of the hospital confirmed that formal ethical review was not required.

### Data Collection

After enrolment, patients were asked to complete a written questionnaire. The compiled questionnaire composed of the modified Fagerström test and other questions were derived from the questionnaire based on the ASE-model of STIVORO, the Michigan Alcohol Screening Test, Jellinek’s self-test on addictions, the DSM V criteria and Compulsive Behavior Questionnaire [27–32]. The questionnaire was tested with cognitive interviewing [33]. Subsequently, we made minor changes that simplified the completion of the questionnaire. The compiled questionnaire is available upon request (English, Dutch). It consisted of 51 questions on general information, current and past smoking, alcohol and addictive drugs use. In this study, we do not report the results on alcohol, drugs and other addictions.

The questions on general information were about educational level, current medication use and any readmission or reoperation since bariatric surgery. Questions on smoking involved the following: current smoking behaviour; any history of smoking; total time of smoking during lifetime, during preoperative period and postoperative period (pack-years); time of preoperative smoking cessation; type and amount of tobacco (cigarette, rolling tobacco, pipe, cigar); amount and duration of longest quit attempt; number and type of used methods at attempt; thoughts about smoking cessation; consequences on health, weight and success of bariatric surgery; reasons to quit; the experienced support by others for cessation; and chance of continuation of cessation in stressful times. Questions on alcohol and addictive drug use involved any use during last week; comparison of current use with the use *before* bariatric surgery; signs of dependency; problems in relations because of use; and fights/hospital admission/detention because of alcohol or drugs.

In addition, preoperative and follow-up data were retrieved from medical records. Preoperative characteristics that were extracted from medical records included sex, preoperative weight, preoperative body mass index (weight in kg/(height in meter)^2^), diabetes mellitus type 2 (DM 2), hypertension, previous abdominal surgery, type and date of bariatric surgery, haemoglobin A1c (HbA1c) and C-reactive protein (CRP). Postoperative characteristics that were collected at the time of questionnaire included remission of hypertension and DM 2, symptomatic gallstones, (history of) reported physical or mental problems due to excess skin (including already performed plastic surgery), reported hypoglycaemia, readmissions related to bariatric surgery or due to abdominal complaints, any reoperation change of HbA1c, change of CRP and % total weight loss (%TWL). Reoperations included laparoscopic cholecystectomy, diagnostic laparoscopy, closure of mesenteric defects (with or without active internal hernia), redo surgery, undo surgery, revisional surgery (for instance, because of bleeding, leakage, stenosis, abscess), surgical neurectomy and incisional hernia repair.

The questionnaires and other data were collected and managed by two researchers (PW and OA). The data were collected and recorded systematically, in accordance with instructions that were formulated in advance. Deviations were discussed until consensus was reached.

### Definitions

Definitions of smoking status were based on behaviour of the last week. *Current smoking* was defined as smoking at least once during the last week. Before surgery, three non-smoking categories were distinguished; ‘never smoker’, ‘former smoker’ (abstinence longer than 6 weeks) and ‘recent smoker’ (abstinence for 1 to 6 weeks). After surgery, we distinguished two non-smoking groups: ‘former smokers’ (abstinence since 1 week or longer) and ‘never smokers’. For the postoperative smoking status, we also specified whether patients smoked continuously after surgery. We reported the behaviour to be ‘continuous’ when the current behaviour was present in ≥ 90% of the postoperative time. Accordingly, we used the term ‘not continuous’ when the behaviour was present in less than 90% of the total time after surgery. Lastly, patients who started smoking after surgery for the first time were called ‘new smokers’. One pack-year was defined as 20 cigarettes or rolling tobacco daily during 1 year. One cigar equalled three cigarettes in case of cigar smoking. One water pipe equalled one cigarette. ‘Addictive drug’ was defined as any addictive prescribed or addictive illicit drugs. Hypertension remission was defined as a blood pressure < 140/90 mmHg after surgery, without using any antihypertensive drug in patients with a history of hypertension before surgery. DM 2 remission was defined as HbA1c <48 mmol/mol after surgery, without any antidiabetic drug in patients with a history of DM 2 before surgery. Educational level was divided into three groups: ‘low’ (primary education, lower vocational education), ‘middle’ (general secondary education, secondary vocational education, higher general and preparatory scientific education) and ‘high’ (higher vocational education and scientific education, scientific education-doctor of philosophy).

### Outcomes Measures

With this study, firstly, we aim to estimate the frequency of current smoking, former smoking and never smoking in patients who underwent bariatric surgery at least 18 months ago.

Secondly, in patients with a history of smoking, we aim to compare questionnaire findings on history and thoughts about smoking cessation between smoking behaviour categories.

We also aim to establish the association between long-term outcomes of bariatric surgery and smoking behaviour categories.

### Statistical Methods

Characteristics of patients before surgery were summarized for all patients and by postoperative smoking behaviour group. Characteristics of patients before surgery, questionnaire findings on smoking history and thoughts about smoking and long-term outcomes of bariatric surgery were compared between the postoperative smoking behaviour groups.

Values are expressed as mean and standard deviation (SD) in case of continuous variables that are normally distributed. Median and interquartile range (IQR) are presented in case of non-normal distribution. Categorical variables are expressed as frequencies and percentages.

Categorical variables were compared using chi-square tests. The independent *t* test (two groups) or one-way ANOVA (more than two groups) were used for normal distributed continuous variables. In case of non-normal distributed data the Mann-Whitney *U* test (two groups) or Kruskal-Wallis test (more than two groups) were used. Finally, in case of *p* value < 0.2, group differences were tested separately using chi-square test, independent *t* test or Mann-Whitney *U* test as appropriate.

The associations between postoperative smoking behaviour categories and long-term outcomes of surgery were explored using univariable logistic or linear regression. Odds ratios (ORs) with 95% confidence interval (95 CI) or betas with standard errors (SE) were presented. Since this evaluation was not the primary aim of the study, we proceeded only to a multivariable model if there was an indication for a relation for the specific outcome (*p* < 0.2). In a multivariable model, variables with a *p* value < 0.4 at univariable analysis were then further explored using multivariable logistic or linear regression (full model). By means of stepwise backward selection, variables with *p* > 0.05 were eliminated from the model (final model).

A *p* value ≤ 0.05 was considered statistically significant. Data analysis was performed using IBM SPSS Statistics software package for Windows version 25 (Chicago, IL).

## Results

### Study Population

A total of 609 (52.0%) patients responded and were included in the study, whereas 557 (47.5%) patients did not respond and 6 (0.5%) patients had died before the inclusion period. Respondents were slightly older than non-respondents (mean (SD) age was 45.9 (9.7) versus 42.2 (10.4) years; *p* < 0.001, respectively), but the remaining characteristics were not significantly different (Table 1).

**Table 1:** Comparison of baseline characteristics between respondents and non-respondents

	Respondents	Non-respondents	*p* value
	*N* = 609	*N* = 556	
Female gender, *N* (%)	522 (85.7)	479 (86.2)	0.830
Age (years), mean (SD)	45.9 (9.7)	42.2 (10.4)	< 0.001
BMI (kg/m)^2^, median (IQR)	42.5 (40.1–46.2)	42.0 (39.7–45.9)	0.150
Hypertension, *N* (%)	242 (39.7)	201 (36.6)	0.275
Diabetes mellitus 2, *N* (%)	119 (19.5)	105 (19.1)	0.858
Previous abdominal surgery, *N* (%)^	328 (53.9)	306 (55.7)	0.521
Primary surgery, *N* (%)	542 (89.0)	477 (86.9)	0.269

N = number; SD = standard deviation; BMI = body mass index; IQR = interquartile range; ^ = including bariatric surgery

Overall, 522 (85.7%) of the patients were female, the mean (SD) age was 45.9 (SD 9.7) years and the median (interquartile range [IQR]) body mass index (BMI) was 42.5 (40.1–46.2) kg/m^2^. Medical history revealed that 242 (39.7%) patients had hypertension, 119 (19.5%) had DM 2 and 328 (53.9%) patients had a previous abdominal surgery, including 67 (11.0% of total population) patients with a previous bariatric surgery. The educational level was reported to be ‘low’ by 54 (9.0%) patients, ‘middle’ by 435 (72.5%) patients and ‘high’ by 111 (18.5%) of all patients.

Table 2 describes and compares the preoperative characteristics of respondents for each smoking behaviour as reported at the time of questionnaire after surgery, separately.

**Table 2:** Demographic and clinical characteristics before surgery, by current smoking behaviour that was reported after surgery

	Current smoker	Former smoker	Never smoker	*p* value
	*N* = 101 (16.6%)	*N* = 239 (39.2%)	*N* = 269 (44.2%)	
Female gender, *N* (%)	87 (86.1)	197 (82.4)	238 (88.5)	0.150
Age (years), mean (SD)	43.0 (9.6)	48.9 (9.2)	44.4 (9.4)	<0.001^ab^
BMI (kg/m)^2^, median (IQR)	42.5 (39.7–46.4)	42.5 (40.1–45.9)	42.5 (40.1–46.2)	0.952
Hypertension, *N* (%)	33 (32.7)	106 (44.4)	103 (38.3)	0.107^a^
Diabetes mellitus 2, *N* (%)	12 (11.9)	65 (27.2)	42 (15.6)	<0.001^ab^
Previous abdominal surgery, *N* (%)^	58 (57.4)	128 (53.6)	142 (52.8)	0.723
Primary surgery, *N* (%)	94 (93.1)	209 (87.4)	239 (88.8)	0.316
Type of surgery, *N* (%)				0.056^c^
RYGB	98 (97.0)	237 (99.2)	268 (99.6)	
Sleeve gastrectomy	3 (3.0)	2 (0.8)	–	
Other	–	–	1 (0.4)	
CRP (mg/l), median (IQR)	7.6 (3.8–12.8)	7.0 (3.6–12.9)	8.7 (4.2–14.7)	0.263
HbA1c (mmol/mol), median (IQR)	38.0(36.0–40.0)	39.0 (36.0–45.0)	37.0 (34.0–41.0)	<0.001^ab^
Tobacco exposure (pack-years*), median (IQR)	15.0 (6.0–22.5)~	15.0 (5.5–30.0)~~	n.a.	0.194
Educational level, *N* (%)				0.709
Low	11 (11.0)	21 (8.9)	22 (8.3)	
Middle	75 (75.0)	171 (72.5)	189 (71.6)	
High	14 (14.0)	44 (18.6)	53 (20.1)	

N = number; SD = standard deviation; IQR = interquartile range; BMI = body mass index; RYGB = Roux-en-Y gastric bypass; CRP = C-reactive protein; HbA1c = haemoglobin A1c; n.a. = not applicable

*One pack-year equals 20 cigarettes (or equivalent amount of tobacco) daily during 1 year;

^^^Including bariatric surgery;

^~^5 missing

^~~^53 missing

^a^significant difference (*p* < 0.05) between current and former smoker

^b^significant difference (*p* < 0.05) between former and never smoker

^c^significant difference (*p* < 0.05) between current en never smoker

### Smoking Prevalence and Smoking Behaviour During Preceding Years

**Fig. 1:**
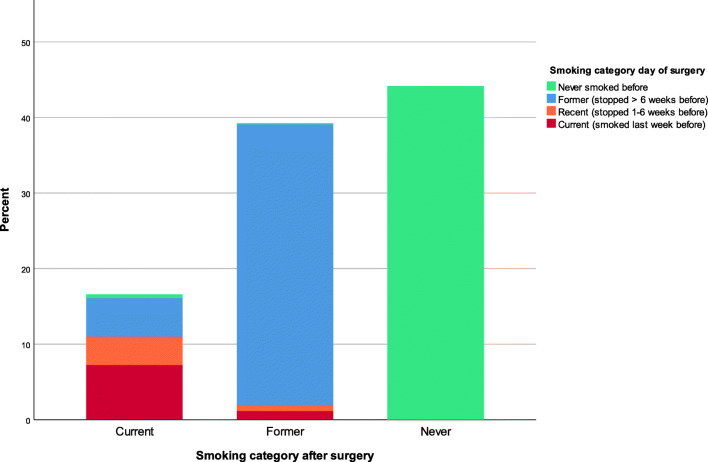
Prevalence of smoking after bariatric surgery, combined with smoking behaviour category on day of surgery. At the time of questionnaire, 101 (16.6%) of the 609 patients reported to be a current smoker; on the day of surgery, 44 of them were current smokers, 23 were recent smokers, 31 preoperative former smokers resumed after surgery and 3 never smokers before surgery started after surgery. Two hundred thirty-nine (39.2%) patients were former smokers. The majority of them (*n* = 226 (94.6%)) was already a former smoker before surgery, 12 patients were recent/current smokers before surgery and one never smoker started and stopped after surgery. Two hundred sixty-nine (44.2%) patients were lifetime never smokers. The groups were significantly different after surgery (chi-square test *p* < 0.001)

Median (IQR) time after surgery was 3.0 (2.3–3.3) years, when patients completed the questionnaire. After surgery, 101 (16.6%) patients were current smokers, 239 (39.2%) were former smokers (226 (94.6%) of them were already former smoker before surgery), and 269 (44.2%) patients were lifetime never smokers. Figure 1 demonstrates the postoperative smoking status, with information on the smoking behaviour category on the day of surgery.

In Fig. 1 and Table 3, the smoking, smoking cessation, resuming and starting rates after surgery are demonstrated.

**Table 3:** Smoking behaviour before and after surgery, as reported after surgery

		Overall	Smoking behaviour after surgery		
		N (%)	Postop current, *N* (%)	Postop former, *N* (%)	Lifetime never, *N* (%)
		609 (100.0)	101 (16.6)	239 (39.2)	269 (44.2)
Smoking behaviour before surgery	Preop current, *N* (%)	51 (8.4)	44 (43.6)	7 (2.9)	n.a.
	Preop recent, *N* (%)	28 (4.6)	23 (22.8)	5 (2.1)	n.a.
	Preop former, *N* (%)	257 (42.2)	31 (30.7)	226 (94.6)	n.a.
	Never smoker, *N* (%)	273 (44.8)	3 (3.0)	1 (0.4)	269 (100)

N = number; postop = postoperative; preop = preoperative; n.a. = not applicable

Overall group differences were tested with chi-square test: *p* < 0.001

### Smoking History in Postoperative Current Smokers

Of the current smokers after surgery, 69 (68.3%) had smoked continuously after surgery (by definition > 90%, median (IQR) 100% (97.8–100); not continuously smokers had smoked median (IQR) 48.0% (15.4–78.3) of the time after surgery.

Postoperative current smokers had a median (IQR) smoking history of 13.4 (5.0–22.3) pack-years; continuous smokers 15.0 (6.0–22.5) years and incomplete continued smokers 9.1 (1.5–20.1) years.

Median (IQR) time of not smoking before surgery was 0.5 (0–2.3) months in postoperative current smokers; 0.25 (0–1) months in those who smoked continuously after surgery; and 2.3 (0.6–24) months in not continuous smokers.

The 54 patients (recent or former smokers before surgery) who resumed smoking after surgery had a median (IQR) history of 10.0 (4.4–20.0) pack-years. Median (IQR) time of not smoking before surgery was 1.8 (0.8–12.0) months. The median (IQR) percentage of time that they had smoked after surgery was 90.6% (47.0–99.4).

### Smoking History in Postoperative Former Smokers

Thirteen (5.4%) of all former smokers had smoked more than 10% of the time after surgery (median (IQR) 73.7% (51.0–92.3)) but were stopped already when they filled in the questionnaire.

Postoperative former smokers had a median (IQR) history of 15.0 (5.0–30.0) pack-years; this was 4.6 (2.4–12.0) pack-years for those who had a temporary relapse after surgery and 15.0 (5.5–30.0) pack-years in those who continued not to smoke anymore. Median (IQR) time of preoperative cessation was 99.0 (31.5–204.0) months; 1.8 (0.5–11.5) months in those who had a temporary relapse and 108.0 (36.0–204.0) months in those who continued not to smoke anymore.

The 12 (5.0%) former smokers who were recent or current smokers before surgery had a median (IQR) history of 10.0 (3.8–17.3) pack-years. Median (IQR) time of cessation before surgery was 0.3 (0.0–0.5) months. They had smoked a median (IQR) of 0.0% (0.0–80.9) of the time after surgery.

### Beliefs About the Effects of Smoking Cessation and Description of Quit Attempts

Table 4 demonstrates the ideas about the health consequences of smoking cessation in postoperative current and former smoking patients (based on combination of pre- and postoperative smoking behaviour). Former smokers were more aware of the beneficial effects of smoking cessation on general health.

**Table 4:** Ideas about smoking cessation and consequences for health in postoperative patients, by postoperative smoking behaviour

	Overall	Current smoker	Former smoker	*p* value
	*N* = 340	N = 101	*N* = 239	
Effect of cessation on general health				< 0.001
Much better	166 (52.4)	20 (20.6)	146 (66.4)	
Better	99 (31.2)	47 (48.5)	52 (23.6)	
Little better	17 (5.4)	11 (11.3)	6 (2.7)	
Not better	8 (2.5)	2 (2.1)	6 (2.7)	
Do not know	27 (8.5)	17 (17.5)	10 (4.5)	
Effect of cessation on result of bariatric surgery				< 0.001
Much better	162 (51.6)	16 (16.5)	146 (67.3)	
Better	49 (15.6)	26 (26.8)	23 (10.6)	
Little better	9 (2.9)	9 (9.3)	0	
Not better	18 (5.7)	12 (12.4)	6 (2.8)	
Do not know	76 (24.2)	34 (35.1)	42 (19.4)	
Effect of cessation on weight loss				<0.001
Better to lose weight	60 (19.2)	4 (4.2)	56 (25.7)	
Not better to lose weight	73 (23.3)	28 (29.5)	45 (20.6)	
Not to lose weight	10 (3.2)	5 (5.3)	5 (2.3)	
Gain weight	22 (7.0)	16 (16.8)	6 (2.8)	
Do not know	148 (47.3)	42 (44.2)	106 (48.6)	
Amount of smokers in social environment				< 0.001
(Almost) none	151 (47.0)	21 (21.2)	130 (58.6)	
Less than half	74 (23.1)	23 (23.2)	51 (23.0)	
50–50	45 (14.0)	20 (20.2)	25 (11.3)	
More than half	30 (9.3)	19 (19.2)	11 (5.0)	
Almost all	21 (6.5)	16 (16.2)	5 (2.3)	

All values are expressed as number with percentages in parentheses

Also, the result of bariatric surgery after smoking cessation was thought to be ‘much better’ by 67% of former smokers versus 17% of current smokers. Characteristics of current and/or previous quit attempts of all current and former smokers at the time of questionnaire after surgery (based on lifetime history of smoking) are described in Table 5.

**Table 5:** Characteristics of (previous) cessation attempts in current and former smokers after surgery

	Overall	Current smoker	Former smoker	*p* value
	N = 340	N = 101	N = 239	
Number of quit attempts, median (IQR)	1.0 (1.0–2.0)	2 (1–3)	1 (1–1)	0.004
Number of used methods to quit, *N* (%)				0.001
0 methods	240 (75.9)	70 (70.7)	170 (78.3)	
1 method	58 (18.4)	16 (16.2)	42 (19.4)	
2 or more methods	18 (5.7)	13 (13.1)	5 (2.3)	
Type of used methods to quit, *N* (%)				^
NRT	29 (30.2)	13 (24.0)	16 (30.8)	
Drugs	28 (29.2)	16 (40.0)	12 (23.1)	
Book/course	12 (12.5)	2 (4.0)	10 (19.2)	
e-Cigarette	7 (7.3)	5 (12.0)	2 (3.8)	
Laser/acupuncture	14 (14.6)	3 (8.0)	11 (21.2)	
General practitioner	6 (6.3)	5 (12.0)	1 (1.9)	
Experienced support with cessation, *N* (%)				< 0.001
A lot	79 (24.6)	12 (12.1)	67 (30.2)	
Medium/moderate	53 (16.5)	30 (30.3)	23 (10.4)	
Little	26 (8.1)	17 (17.2)	9 (4.1)	
None	45 (14.0)	18 (18.2)	27 (12.2)	
Not applicable	118 (36.8)	22 (22.2)	96 (43.2)	
Continuation of cessation in stressful situation, *N* (%)				< 0.001
Definitely not	38 (11.8)	30 (30.6)	8 (3.6)	
Probably not	33 (10.3)	32 (32.7)	1 (0.4)	
Neutral/do not know	29 (9.0)	18 (18.4)	11 (4.9)	
Probably yes	16 (5.0)	9 (9.2)	7 (3.1)	
Certainly yes	205 (63.9)	9 (9.2)	196 (87.9)	
Planning of cessation in future, *N* (%)			n.a.	n.a.
Within 1 month		14 (17.7)		
Within 6 months		28 (35.4)		
Within 12 months		15 (19.0)		
Within 5 years		1 (1.3)		
Not within 5 years		3 (3.8)		
Never planning to quit		18 (22.8)		
Main reason (for plan) of cessation^$^, *N* (%)				0.003
Request doctor	24 (9.5)	9 (11.8)	15 (8.5)	
Consequences health	167 (66.3)	45 (59.2)	122 (69.3)	
Costs	20 (7.9)	13 (17.1)	7 (4.0)	
Request family/friends	27 (10.7)	4 (5.3)	23 (13.1)	
Taste/smell	14 (5.6)	5 (6.6)	9 (5.1)	
Main reason (for plan) of cessation^&^, *N* (%)				0.001
Request doctor	30 (11.9)	12 (15.8)	18 (10.2)	
Consequences health	147 (58.3)	37 (48.7)	110 (62.5)	
Costs	21 (8.3)	14 (18.4)	7 (4.0)	
Request family/friends	33 (13.1)	7 (9.2)	26 (14.8)	
Taste/smell	21 (8.3)	6 (7.9)	15 (8.5)	

N = number; IQR = interquartile range; NRT = nicotine replacement therapy; n.a. = not applicable

^^^No comparison between subgroups because of multiple reported methods per patient

^$^Most internal motivation

^&^Most external motivation

### Outcomes of Bariatric Surgery and Smoking Behaviour

Table 6 shows the long-term bariatric outcomes per smoking behaviour category after surgery (based on lifetime smoking history). Mean %TWL in current smokers was 33.6 (SD 8.9). %TWL was 3.4% (SE 1.0) higher in current smokers compared with former smokers and 2.1% (SE 1.0) higher compared with lifetime never smokers (overall *p* < 0.001). In a multivariable model with adjustments for sex, preoperative BMI, HbA1c before surgery and time after surgery, %TWL in current smokers was 2.8% (SE 0.9) higher compared with former smokers and 2.1% (SE 0.9) higher compared with never smokers (overall *p* = 0.011).

**Table 6:** Long-term outcomes, by smoking status after surgery

	Total	Postop current smoker	Postop former smoker	Never smoker	*p* value
	N = 609	N = 101	N = 239	N = 269	
% TWL, mean (SD)	31.3 (8.3)	33.6 (8.9)	30.2 (8.3)	31.5 (7.8)	0.002^$^
Hypertension remission, *N* (%)	135 (55.8)	20 (60.6)	62 (58.5)	53 (51.5)	0.495
DM 2 remission, *N* (%)	51 (42.6)	6 (50.0)	29 (44.6)	16 (38.1)	0.697
% HbA1c change, median (IQR)	−6.4 (−13.2 to 0.0)	−3.1 (−13.0 to 0.0)	−8.3 (−15.6 to 0.0)	−5.6 (−11.9 to 0.0)	0.017^#^
Reported hypoglycaemia, *N* (%)	36 (6.0)	8 (8.2)	10 (4.2)	18 (6.7)	0.294
Readmissions abdominal complaints, *N* (%)	144 (24.0)	27 (27.0)	56 (23.8)	61 (23.0)	0.727
Symptomatic gallstones, *N* (%)	68 (11.2)	14 (13.9)	25 (10.5)	29 (10.8)	0.653
Reoperation during follow-up, *N* (%)	106 (17.7)	23 (23.0)	38 (16.2)	45 (17.0)	0.304
Hindrance of excess skin (or already corrected), *N* (%)	170 (27.9)	27 (26.7)	61 (25.5)	82 (30.5)	0.442
% CRP change, median (IQR)	−84.3 (−92.2 to − 71.5)	−86.7 (−92.0 to − 75.1)	−82.5 (−91.6 to − 66.4)	−85.2 (−93.2 to − 73.2)	0.156

Postop = postoperative; *N* = number; TWL = total weight loss; SD = standard deviation; DM 2= Diabetes mellitus 2; HbA1c = haemoglobin A1c; IQR = interquartile range; CRP = C-reactive protein

^Including bariatric surgery

^$^Significant difference between current and former smokers (*p* = 0.001) and between current and never smokers (*p* = 0.028)

^#^Significant difference between former and never smokers (*p* = 0.012) and between current and former smokers (*p* = 0.017)

Median % HbA1c change was similar in the three groups, with somewhat more decrease in former smokers compared with never and current smokers (*p* = 0.013); median (IQR) absolute HbA1c decrease was 3.0 (0.0–7.0), 2.0 (0.0–5.0) and 1.0 (0.0–5.0) mmol/mol, respectively. In the multivariable analysis, where we adjusted for preoperative HbA1c and revisional surgery, the difference was not statistically significant.

Remission of DM 2, remission of hypertension, reported hypoglycaemia, readmissions because of abdominal complaints, reoperations related to bariatric surgery, symptomatic gallstones and reported physical or mental hindrance of excess skin were not statistically different between the groups. Univariable analysis of smoking subgroups and CRP change showed an indication to proceed to multivariable analysis. However, in the multivariable model, the association was not statistically significant.

## Discussion

This is the first study in bariatric surgery evaluating self-reported smoking behaviour combined with thoughts about the health consequences of smoking cessation and actual health outcomes in current, former and never smokers. In this study, the prevalence of smoking after surgery was 16.6% and 44.2% of the patients had never smoked. Nineteen percent of the patients that were ex-smokers (recent or former smokers) before surgery resumed smoking after surgery. Of the patients that currently smoked or recently smoked before surgery, 15% stopped after surgery. Compared with postoperative current smokers, former smokers were more aware of the beneficial effects of smoking cessation on the general health and the result of the bariatric surgery. Some of them even expected an unrealistic positive effect of smoking cessation on weight loss. Actual mean weight loss was somewhat more in smokers compared with former and never smokers. We could not confirm that other long-term bariatric outcomes were significantly different between current, former and never smokers after surgery.

Previous studies describing and associating smoking behaviour before and after bariatric surgery have several methodological limitations [34]. Timing of registration and definitions of smoking were dissimilar, rough and/or missing; studies had small sample sizes; high percentages of loss-to-follow-up and inclusion of types of surgery were often unclear. This could explain why the ranges of smoking prevalences are divergent, before and after bariatric surgery (1.2–62.0% and 8.1–43.3%, respectively) [23, 25, 31, 35, 36]. Also, it could (partially) explain why studies investigating the association of smoking with TWL (and other bariatric surgery outcomes) have conflicting results [7, 20–23, 25, 35–37].

Theoretically, the increased TWL in relation to smoking could be explained mainly by the effect of nicotine on (1) energy expenditure, (2) appetite/satiety and (3) eating behaviour (motivational and emotional influences). The absence of nicotine may cause reduced energy expenditure, more appetite and different eating behaviour. Furthermore, after smoking cessation, the rewarding value of food increases, which induces intake of greater amounts of sugar and fat, achieving pleasure similar to that of smoking [38]. One study found that weight gain was less in patients who were treated with cognitive-behavioural therapy focussing on the *concerns about the weight gain* after cessation, as opposed to the patients who were treated with strategies to reduce the weight gain itself [39]. The weight gain after smoking cessation is on average 4.5 kg in the general population and occurs especially during the first 6 months; 13% of quitters gain more than 10 kg. Lastly, two studies showed relatively higher smoking prevalences more than 6.5 years after surgery (27.5–43.0%), possibly due to the typical relapse pattern of smoking addiction, or because patients use smoking for weight control when the normal slope of weight gain after surgery begins. [13, 22, 35, 37, 38] The time after surgery in our study was too short to confirm these findings.

The analyses of associations of postoperative smoking with the other clinical outcomes showed no significant differences. Remarkably, compared with former and never smokers, the prevalences of DM 2 and hypertension in current smokers were already relatively low before surgery. The observed differences in TWL and number of cases in the subgroups are probably too subtle to translate in detectable changes in metabolic regulation, symptomatic gall stones and other clinical outcomes after surgery. And, although we were not able to statistically verify previously described relations with smoking and postoperative comorbidities, we are still convinced that smoking cessation should be promoted.

### Beliefs About Smoking and Health Risks Among Smokers and Ex-Smokers

This is the first study exploring the attitudes of smokers and ex-smokers toward benefits of smoking cessation and risks of smoking in bariatric surgery patients. The attitudes and beliefs have been investigated in other populations [12–19]. We could imagine that the success and willingness to quit are affected by several aspects of which the concern of weight gain after smoking cessation is one, both before and after bariatric surgery. Other factors that could give impact on the willingness to quit are of a social and cultural nature; as in our study we found differences in the experienced support and amount of smokers in social environment between former and current smokers. Future (qualitative) research could deepen the knowledge about (former) smokers, in respect of the contributing or interfering beliefs and factors associated with successful cessation in bariatric surgery populations. Psychosocial theories are used to investigate behavioural change, and smoking cessation in particular [14–19, 40, 41]. In general, smokers are more likely to deny or rationalize their risk of developing smoking-related diseases (self-exempting beliefs). When smokers experience enough cognitive dissonance, this could discomfit the self-exempting beliefs, thereby inducing smoking cessation attempt. [17, 41]. This could explain the differences in beliefs between smokers and former smokers about the effects of smoking cessation on general health, bariatric surgery and weight loss. The proportion of responses of ‘do not know’ can be explained in several ways; respondents truly did not know, or they were not able to figure it out due to cognitive dissonance. Some factors are known to be associated with poor self-reported health rate, including smoking and, among other things, BMI, gender, age and educational level [42, 43]. It is possible that these factors also influence the attitudes toward health effects of smoking cessation, but we did not adjust for them.

### Limitations

Our study has several limitations. First of all, self-reported smoking can be subject to recall and reporting bias [12, 44]. It is known that reporting bias is greater in situations where quitting expectations on part of the healthcare team are higher [44]. In our experience, these biases are highest before surgery, because continuation of smoking could result in postponement of the surgery. In this study, we used written self-report without interference of the attending doctor. Thus, the (retrospective) design could have been a positive aspect, because patients did not have to fear rejection when they retrospectively reported that they were a current smoker at the day of surgery. Additionally, we previously demonstrated that the sensitivity and specificity of written self-reported smoking behaviour compared with serum cotinine in patients more than 18 months after bariatric surgery were 93.5% and 96.4% [34]. Thus, we expect that the contamination of the ‘former smoker category’ with smokers that reported their smoking status incorrectly was limited. However, associations with smoking could actually be more distinct when we could have prevented any misclassification. Second, the response rate was only 52%. Apparently, the bariatric surgery population is not easily willing to participate in studies concerning smoking behaviour, considering most of the response rates in other questionnaire studies concerning bariatric surgery and smoking were even lower or unclear [21, 24, 31]. Additionally, the percentages of loss-to-follow-up after more than 2 years in bariatric surgery patients are high (26–95.7%), even in studies involving only medical record review, often above 40% [7, 23, 45, 46]. We compared preoperative characteristics of responders and non-responders to check for selection bias, which seems limited. Still, the response rate could influence the representativeness of the data. Patients with specific characteristics may have systematically refused to participate, i.e., based on smoking, the most (un)satisfied, (un)healthy or optimistic patients could have ignored our request to participate. Therefore, we interpret the results with caution, because attrition bias could affect the magnitude and even direction of the associations that we found. Because of the possibility of Berkson’s bias, current findings cannot be externalized to other populations and should first be examined in other bariatric surgery populations.

Third, the questionnaire was developed specifically for this study and not validated. However, we carefully selected questions used in the previous publications and in clinical practice. Also, we used cognitive interviewing to improve the formulating of the questions. Therefore, we are convinced that we can draw reliable and clinically relevant conclusions. Lastly, we included a heterogeneous group in terms of type of bariatric procedures and primary and revisional surgery, which could have influenced our results. On the other hand, in terms of preoperative assessment and postoperative follow-up duration and method, this group was uniform.

In conclusion, 3 years after bariatric surgery, 16.6% of the patients was smoking, and 44.2% had never smoked in their entire life. Former smokers were more aware of the beneficial effects of smoking cessation on the general health and the result of the bariatric surgery compared with current smokers. Actual mean weight loss was somewhat more in smokers compared with former and never smokers. We could not confirm that other long-term bariatric outcomes were significantly different between current, former and never smokers after surgery. The present study emphasizes the need for larger cohort studies with long-term follow-up, investigating better pre- and postoperative strategies to convince smokers to quit and prevent former smokers to relapse. Future research should focus on these strategies, possibly by addressing concerns about weight gain and other reasons interfering with quitting.
